# Caregivers’ Perceptions, Needs, and Data Sharing Concerns in mHealth Research on Pediatric Asthma: Cross-Sectional Survey Study

**DOI:** 10.2196/49521

**Published:** 2023-12-19

**Authors:** Glen Meng, Maliha Jan Ali, Sze Man Tse

**Affiliations:** 1 Faculty of Medicine, Université de Montréal, Montréal, QC, Canada; 2 Division of Respiratory Medicine, Department of Pediatrics, Centre Hospitalier Universitaire Sainte-Justine, Montreal, QC, Canada

**Keywords:** asthma, mHealth, mobile health, app, application, apps, applications, pediatrics, caregivers, knowledge translation, cross-sectional, survey, surveys, respiratory, pulmonary, lung, pediatric, data sharing, information sharing, privacy, usability, confidentiality, child, children, caregiver, caregiving, patient knowledge

## Abstract

**Background:**

Pediatric asthma is the most common chronic respiratory disease of childhood. Caregivers often report lacking knowledge in several aspects of asthma management at home. Although the use of mobile health (mHealth) tools, such as mobile apps, could facilitate asthma self-management and, simultaneously, the collection of data for research, few studies have explored the features that caregivers would like to see in such a tool and their perceptions on data sharing.

**Objective:**

This study evaluates caregivers’ perceived knowledge gaps in asthma management; their perceptions of certain features and resources that should be included in a potential mobile app; and any concerns that they may have regarding data sharing for research, including privacy and security concerns.

**Methods:**

In this cross-sectional study, we surveyed 200 caregivers of children (aged 1-13 y) with asthma who were followed at a pediatric tertiary care center in Montreal, Canada. Anonymous data were collected through the institutional web-based survey platform. We collected the participants’ answers by using a 5-category Likert scale (“completely agree,” “agree,” “neither agree nor disagree,” “disagree,” and “completely disagree”), multiple-choice questions, and free-text questions on the abovementioned topics. Descriptive statistics were performed, and answers were compared between caregivers of preschool-aged children and caregivers of school-aged children.

**Results:**

Participating children‘s mean age was 5.9 (SD 3.4) years, with 54% (108/200) aged ≤5 years and 46% (92/200) aged >6 years. Overall, caregivers reported having adequate knowledge about asthma and asthma self-management. Nonetheless, they identified several desirable features for a mobile app focused on asthma self-management. The most frequently identified features included receiving alerts about environmental triggers of asthma (153/199, 76.9%), having videos that demonstrate symptoms of asthma (133/199, 66.8%), and being able to log children’s asthma action plans in the app (133/199, 66.8%). Interestingly, more caregivers of preschool-aged children preferred textual information when compared to caregivers of school-aged children (textual information for explaining asthma: *P*=.008; textual information for the symptoms of asthma: *P*=.005). Caregivers were generally highly in favor of sharing data collected through a mobile app for research.

**Conclusions:**

Caregivers of children with asthma in our study identified several desirable educational and interactive features that they wanted to have in a mobile app for asthma self-management. These findings provide a foundation for designing and developing mHealth tools that are relevant to caregivers of children with asthma.

## Introduction

Asthma is a chronic pulmonary inflammatory disorder that affects more than 262 million people of all ages around the world [1], imposing a great burden on patients and society. Among all individuals with asthma, children have the highest rates of asthma-related emergency department visits [2]. Furthermore, asthma can persist until adulthood and has been associated with reduced long-term lung function [3,4]. Therefore, the importance of early diagnosis and management lies in the potential maximization of lung function and reduction of asthma-related morbidity [5]. Moreover, optimal asthma self-management can help prevent unscheduled care, symptom exacerbations, and school and work absences, in addition to helping improve the overall quality of life.

One aspect of optimal asthma self-management is having the necessary knowledge and ability to recognize symptoms. Several studies have explored parents’ perceptions of their children’s asthma and the gaps in their knowledge of the pathology and management of asthma [6-9]. Although targeted education is delivered by asthma educators and health professionals, several gaps remain in caregivers’ knowledge of their children’s asthma, which have resulted in many caregivers believing myths about asthma, such as the long-term usage of corticosteroids having a negative impact on cardiac function and dependence [10]. Parents also feel a psychological weight as the caregivers and are often hypervigilant due to the unpredictability of the symptoms of asthma exacerbation [11].

There is thus a need for an effective and accessible tool that can support caregivers in the management of their children’s asthma, particularly in between visits with their health professionals. Information-based technologies can serve as good tools for enhancing parents’ knowledge of their children’s asthma, thereby enabling them to better manage the condition [12]. In particular, the omnipresence of mobile and wireless technologies has driven changes not only in society’s daily life but also in the health care system. In 2017, health apps were downloaded about 3.7 billion times [13], with more than 325,000 health apps available across mobile platforms. *Mobile health* (mHealth) is a term that refers to the “use of mobile phones and other wireless technology in medical care” [14]. Since its introduction into the health care system, many studies evaluating the effects of mHealth have mostly reported positive results with regard to the promotion of self-management, though most of these studies focus on adult patients. For example, a study that was conducted in adult outpatient asthma clinics showed that mobile-based interactive intervention improves pulmonary function, quality of life, asthma symptoms, and treatment adherence while reducing rates of acute exacerbations [15]. mHealth technologies can also help health professionals track patients’ progress and symptoms in order to adapt treatment plans, which can in turn reduce adverse outcomes. mHealth benefits have also been studied for nonrespiratory chronic conditions; such studies demonstrated that mHealth technologies were mostly positively rated in terms of usefulness in the management of the condition, as they increased the patients’ autonomy by making them actors in their own health [16,17].

Although mHealth is already serving a large population, pediatric conditions, including childhood asthma, are commonly neglected when it comes to mHealth technology [18]. Furthermore, the needs of caregivers of children with asthma and whether these needs differ across children of different ages are unknown. Additionally, we could not identify existing mHealth apps for pediatric asthma that explicitly mention that caregivers’ perceptions and needs were taken into account during app development. In order to develop a useful tool for caregivers, it is important to explore the gaps that they believe are present in their knowledge or in their daily routine (ie, those that prevent them from achieving optimal asthma management).

In this study, we assessed caregivers of children (aged 1-13 y) with asthma and their perspectives on using mHealth to facilitate asthma management. Specifically, we surveyed caregivers on their perceived knowledge gaps in asthma management, their perceptions of certain features and resources that should be included in a potential mobile app, and any concerns that they may have regarding data sharing specifically for research. This study provides a foundation for designing and cocreating future mHealth tools for childhood asthma self-management.

## Methods

### Study Design

We conducted a cross-sectional survey of caregivers of children with asthma who were followed at the Centre Hospitalier Universitaire (CHU) Sainte-Justine in Quebec, Canada. This study was conducted from September 1, 2019, to July 1, 2020.

### Ethical Considerations

This study was approved by the CHU Sainte-Justine Research Ethics Board (project number: 2020-2664). Informed consent was obtained with a research information and consent form, which was administered to and signed by participants at the beginning of the survey. Submitted data were anonymous.

### Participants and Procedures

We included the caregivers of (1) children aged 1 to 13 years, inclusively, and (2) children with physician-diagnosed asthma or suspicion of asthma. There were no exclusion criteria. We identified eligible patients from the respiratory medicine and asthma clinics, inpatient units, and pulmonary function test laboratory of the CHU Sainte-Justine. Eligible families were approached in person or contacted by phone, and they were asked to fill in the web-based survey.

### Data Collection and Questionnaire

The survey was administered via the CHU Sainte-Justine LimeSurvey platform (LimeSurvey GmbH). We asked participants to fill in a questionnaire to assess the caregivers’ (1) knowledge on asthma pathogenesis, symptom recognition, and controller and rescue medication use; (2) perceptions on features that they would like to see in a mobile app for facilitating asthma self-management; and (3) openness to using a mobile app for research purposes and their concerns. We constructed the survey based on the themes we identified as important in the mHealth and asthma fields (knowledge gaps, desirable features, mHealth use in research, and data sharing concerns). These themes were based on existing literature and the gaps we identified. We collected the participants’ answers by using a 5-category Likert scale (“completely agree,” “agree,” “neither agree nor disagree,” “disagree,” and “completely disagree”), multiple-choice questions, and free-text questions. We also collected data on the children’s age, sex, and previous asthma history (hospitalizations and emergency department visits in the past year).

### Sample Size and Data Analysis

Our target sample size was 200 caregivers, which would allow for the description of responses with a 7% margin of error at a 95% confidence level. We performed a descriptive analysis of the participants’ baseline characteristics and each item in the questionnaire, using frequencies and percentages. Additionally, we compared the perceptions and needs of caregivers of preschool-aged children to those of caregivers of school-aged children, using the chi-square test for proportions and the nonparametric Kolmogorov-Smirnov test for distributions, such as Likert scale distributions.

## Results

### Participants

A total of 200 caregivers completed the survey, although some questions were left unanswered by some participants. The mean age of the children for whom the parents answered the survey was 5.9 (SD 3.4) years, and most children were male (142/200, 71%)—a proportion that reflects the sex distribution of pediatric asthma in the general population (Table 1). The majority of children were not hospitalized over the past year (146/199, 73.4%), but more than half reported at least 1 asthma-related emergency department visit over the past year (106/199, 53.3%).

**Table 1.** Baseline characteristics of children for whom the parents answered the survey (N=200).

Participant characteristics		Value
Age (y), mean (SD)		5.9 (3.4)
**Age category, n (%)**		
	Preschool-age (≤5 y)	108 (54)
	School-age (6-13 y)	92 (46)
**Sex, n (%)**		
	Male	142 (71)
	Female	58 (29)
**Number of asthma-related emergency department visits in the past year, n (%^a^)**		
	None	93 (46.7)
	1	38 (19.1)
	2	28 (14.1)
	≥3	40 (20.1)
**Number of asthma-related hospitalizations in the past year, n (%^a^)**		
	None	146 (73.4)
	1	31 (15.6)
	2	14 (7)
	≥3	8 (4)

a Percentages were calculated with a denominator (N) of 199, as 1 caregiver did not answer the question.

### Caregivers’ Perceived Asthma Knowledge

**Figure 1.**
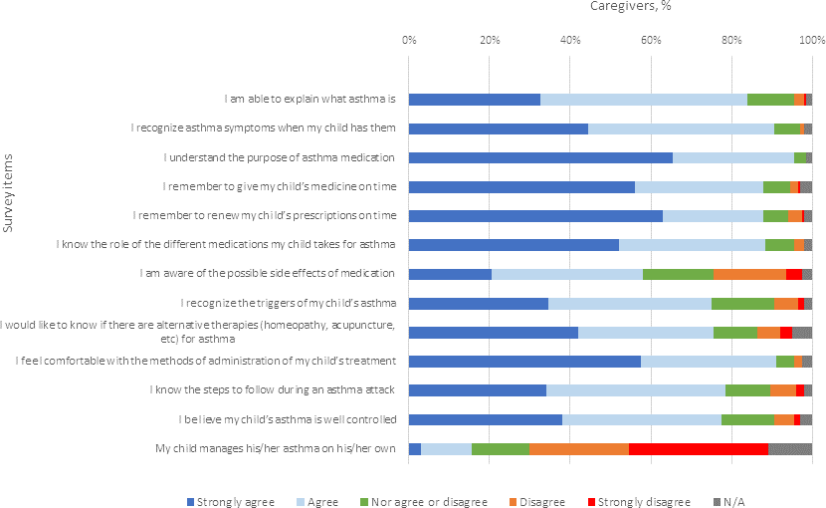
Caregivers’ perceived asthma knowledge. N/A: not applicable.

We first assessed the caregivers’ perceived knowledge on asthma and asthma management (Figure 1). The majority of caregivers strongly agreed or agreed that they understood the purpose of asthma medication (191/197, 97%), could explain what asthma is (168/197, 85.3%), and could recognize asthma symptoms (181/196, 92.3%). Caregivers reported that they remembered to give their children’s medicine on time (176/194, 90.7%), renewed their prescriptions (176/196, 89.8%), and knew the steps to follow during an asthma exacerbation (157/196, 80.1%). In terms of asthma management, most participants reported that they would be able to identify the triggers of their children’s asthma (150/196, 76.5%). Caregivers were comfortable with the methods of administration of their children’s treatment (182/200, 91%) and believed that their children’s asthma was well controlled (155/200, 77.5%). Interestingly, a big proportion of caregivers were curious about whether there were alternative therapies for asthma (151/200, 75.5%), such as homeopathy and acupuncture. As expected for this study’s sample of young children, few children managed their asthma on their own.

### Desirable Features in a Mobile App Focused on Asthma Self-Management

We asked caregivers to identify mobile app features that would be helpful in managing their children’s asthma and would address their needs (Table 2). Several options were listed (more than 1 could be chosen), along with a free-text option. Receiving alerts about environmental triggers of asthma, such as excessive heat and high pollen levels, was the most frequently identified feature (153/199, 76.9%). Caregivers were also interested in videos demonstrating the symptoms of asthma (133/199, 66.8%) and in the personalization of the app to their own children’s situations, which included logging their children’s asthma action plans in the app (133/199, 66.8%). Other features that caregivers perceived as useful were charts of their children’s symptoms (131/199, 65.8%), a short questionnaire that could be completed regularly to monitor asthma symptoms (123/199, 61.8%), information on new asthma studies (121/199, 60.8%), and reminders to renew inhalers (116/199, 58.3%). Less commonly identified app features among caregivers were textual information that explains inhaler techniques (66/199, 33.2%) and what asthma is (71/199, 35.7%) and a platform to interact with other parents of children with asthma (77/199, 38.7%).

**Table 2.** Desirable features in a mobile app for asthma self-management identified by caregivers (N=199).

Features^a^	Caregivers, n (%)
Alerts about asthma triggers in the environment (eg, excessive humidity, high pollen count, etc)	153 (76.9)
Video demonstrating the symptoms of asthma (indrawing, wheezing, etc)	133 (66.8)
The ability to log children’s asthma action plans in the app	133 (66.8)
Charts of children’s asthma symptoms	131 (65.8)
A questionnaire (5-7 questions) to be completed regularly in order to be able to monitor children’s asthma symptoms	123 (61.8)
Information on new studies on asthma	121 (60.8)
Reminders to renew pumps	116 (58.3)
Symptom diary (to be completed manually)	113 (56.8)
Reminders to administer the pumps every day	106 (53.3)
Text explaining the symptoms of asthma	99 (49.7)
Video demonstrating the techniques of pumps’ usage	85 (42.7)
Video explaining what asthma is	83 (41.7)
Internet links for more information on asthma	78 (39.2)
A platform to interact with other parents of children with asthma	77 (38.7)
Text that explains what asthma is	71 (35.7)
Text explaining the techniques of pumps’ usage	66 (33.2)

a More than 1 feature could be chosen by caregivers.

Participants also suggested other needs that were not included in the questionnaire, notably the need for a mobile app adapted to older children and adolescents that could help them develop skills for managing their own asthma and medications. Other caregivers also mentioned the need for a reminder system for upcoming medical appointments.

Stratifying the answers as those from caregivers of preschool-aged children (≤5 y) and those from caregivers of school-aged children (>5 y) showed differences that were statistically significant (Table 3). Specifically, more caregivers of preschool-aged children with asthma than caregivers of school-aged children deemed that a symptom diary (to be completed manually; *P*=.04), text that explains what asthma is (*P*=.008), and text that explains the symptoms of asthma (*P*=.005) would be useful in a mobile app.

**Table 3.** Desirable mobile app features identified by caregivers of preschool-aged children (≤5 y) and school-aged children (>5 y).

Feature	Caregivers of preschool-aged children (n=108), n (%)	Caregivers of school-aged children (n=92), n (%)	*P* value
Questionnaire to be completed regularly to monitor children’s asthma symptoms	72 (66.7)	51 (55.4)	.16
Symptom diary	69 (63.9)	44 (47.8)	.04
Charts of children’s asthma symptoms	70 (64.8)	61 (66.3)	.86
Reminders to administer the pumps every day	58 (53.7)	48 (52.2)	.99
Reminders to renew pumps	65 (60.2)	51 (55.4)	.66
Text that explains what asthma is	48 (44.4)	23 (25)	.008
Text explaining the symptoms of asthma	64 (59.3)	35 (38)	.005
Text explaining the techniques of pumps’ usage	40 (37)	26 (28.3)	.27
Video explaining what asthma is	46 (42.3)	37 (40.2)	.90
Video demonstrating the symptoms of asthma	77 (71.3)	56 (60.9)	.19
Video demonstrating the techniques of pumps’ usage	47 (43.5)	38 (41.3)	.91
The ability to log children’s asthma action plans in the app	75 (69.4)	58 (63)	.48
Alerts about environmental asthma triggers	83 (76.9)	70 (76.1)	.99
Internet links for more information on asthma	44 (40.7)	34 (37)	.73
Information on new studies on asthma	70 (64.8)	51 (55.4)	.26
Platform to interact with other parents of children with asthma	46 (42.6)	31 (33.7)	.28

### Using Personal Data Through mHealth for Research

**Figure 2.**
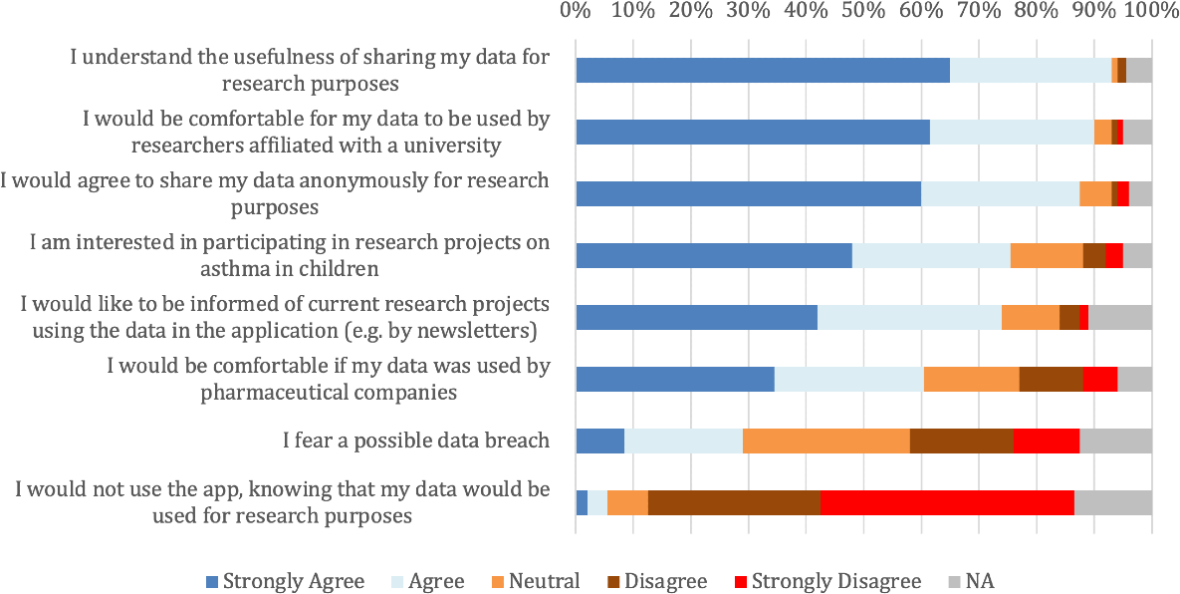
Caregivers’ perceptions on the use of data through mHealth for research. N/A: not applicable.

We evaluated the caregivers’ perceptions on sharing personal data, that is, those entered into a mobile app, with researchers via a secure portal, prefacing that the data would be used to create a database that would allow researchers to carry out projects that aim to improve the management of asthma in children (Figure 2). Most caregivers understood the usefulness of sharing their data for research purposes (186/200, 93%). The majority were comfortable with their data being used by researchers (180/200, 90%) and reported that they would agree to share their data anonymously for research purposes (175/200, 87.5%). Most caregivers were also interested in participating in asthma research projects (151/200, 75.5%) and reported that they would like to be informed of research projects using their data through the app (148/200, 74%). Few caregivers deemed data breach possibilities (58/200, 29%) and the fact that their data would be used for research purposes (11/200, 5.5%) as deterrents for using the mobile tool. We also compared caregivers’ perceptions on the use of personal data for research between caregivers of school-aged children and caregivers of preschool-aged children, which did not show statistically significant between-group differences (*P* values for all responses were <.05).

## Discussion

### Principal Results

Our study identified features that caregivers of children with asthma would like to see in an mHealth tool, specifically a mobile app, for asthma management despite their perception of having good knowledge about many aspects of asthma. The highly desirable features have personalizable or interactive components. Additionally, we found that some desired features differed between caregivers of preschool-aged children and caregivers of school-aged children. We also found that the use of data collected through a mobile app for research was highly acceptable among caregivers.

### Comparison With Prior Work

Previous studies documented that caregivers perceived lacking knowledge about asthma pathogenesis [6-8], the medications used in their children’s asthma plans [8], the main triggers of asthma [9] and how to avoid them, and recommended changes that they should make at home and in their children’s environment to avoid any exacerbations [9]. The perception of lack of knowledge can lead to suboptimal adherence [9], which can result in adverse health events. Our study contrasts with previous literature, as most caregivers reported having good knowledge of asthma. This contrast may be related to the nature of our sample, which consisted of caregivers of children who were followed at a tertiary pediatric care center. However, the caregivers still identified several educational features that they would like to see in a mobile app for asthma. For example, while 92.3% (181/196) of caregivers strongly agreed or agreed that they could recognize asthma symptoms when their children exhibited them, 66.8% (133/199) reported that they would like to have a video explaining asthma symptoms in the mobile app. This discordance suggests that in addition to identifying knowledge gaps, studies should directly assess caregivers’ perspectives on their needs and their proposed solutions.

Interestingly, a majority of caregivers wanted information on alternative therapies for the management of asthma (151/200, 75.5%). This corroborates the existing literature; one study found that parents who wanted to explore alternative therapies denoted that health care professionals had little or no knowledge of them [19]. Although there is currently no evidence-based alternative therapy for asthma, our findings highlight that this is an important asthma management–related issue to discuss with caregivers.

Few mobile apps target caregivers of children with asthma or the pediatric population. Although mHealth can be useful in improving health outcomes in the pediatric population, interventions produce larger changes when caregivers are involved rather than when only children are targeted [20]. Thus, it is essential to assess the caregivers’ needs when designing a mobile tool to ensure its relevance. In a study on parents’ and clinicians’ preferences for a mobile app focused on asthma self-management in adolescents, parents identified the ability to input symptoms into a diary and the ability to generate reports for the physician as the most useful features [21]. Reminder messages for medication and prescription renewals were also features that were appreciated by parents, as they encouraged medication adherence. Although the app targeted adolescents, the study gave an overview of parental needs. In our study, several desirable features were identified by caregivers for an app that would target them. These included reminders for medication administration and renewal, environmental alerts that are relevant to asthma, the ability to log symptoms and children’s asthma action plans, and evaluations of asthma control. Many of these features can only be integrated in a mobile or digital tool and cannot be made available through traditional educational sessions or paper materials, underlining the potential usefulness of an mHealth tool. One common theme across these features is the desire for interactivity, which is a key component of mHealth. A second theme is improving asthma management by tracking symptoms and being aware of potential triggers. mHealth tools can readily respond to these needs by providing real-time alerts and feedback to caregivers and by sending programmed medication-related reminders.

Interestingly, we found that some desired features differed between caregivers of preschool-aged children and caregivers of school-aged children, mostly with regard to the mode of education information delivery. More caregivers of preschool-aged children reported that they would like to see textual information on asthma. Although we could not find existing literature to explain this difference in asthma or chronic disease education, our findings suggest that the mode of knowledge transmission is important and has to be considered while creating a mobile tool.

In addition to facilitating knowledge sharing and tracking health, mHealth can be a powerful tool for research. With the user’s consent, information entered into an app can provide valuable, real-world, longitudinal data on patients with different conditions. For example, these data can be used to conduct observational studies on predictors of adverse outcomes, which in turn could result in targeted interventions and improved management. Our findings suggest that caregivers welcomed research projects that are conducted through a mobile app and were open to sharing their data for research. The majority of caregivers were not deterred by a fear of a possible data breach (142/200, 71%) and were comfortable with sharing data with industrial researchers (156/200, 78%). These perspectives can be leveraged to design research projects around patient-centered or caregiver-centered mobile tools.

### Strengths and Limitations

The relatively large sample size of our study allowed us to capture a variety of opinions accurately. Additionally, by including caregivers of preschool-aged children and caregivers of school-aged children, we were able to compare the needs of different populations. Our study also has noteworthy limitations. First, our study surveyed caregivers of children who were followed or treated at a tertiary care center, which may limit the generalizability of our results to children with milder asthma. However, this study population also represents the sickest children with asthma, who may benefit the most from achieving improved self-management through a mobile tool. Second, we assessed only the caregivers’ perceptions of asthma knowledge. Caregiver knowledge gaps have been extensively documented in the literature. Although this was not within the scope of this study, these knowledge gaps should be also be addressed when creating an educational tool. Third, given the lack of a standardized survey for assessing parental perceptions of mHealth in asthma management, we constructed our survey based on the knowledge gaps that we identified based on existing literature and our team’s clinical experience. Although we checked survey responses for errors and consistency (eg, between items such as “I understand the purpose of asthma medication” and “I know the role of the different medications my child takes for asthma”), the psychometric properties of the survey have not been formally evaluated, and some specific topics may not have been addressed (eg, the use of graphical information). Fourth, given the design of this study, we did not conduct qualitative interviews with the parents, which could have provided a more complete understanding of parental perceptions and concerns. Finally, we did not assess the preferences and needs of adolescents. As they represent a distinct population and are more likely to independently self-manage their asthma, it is important to assess their needs through a survey that specifically targets them.

### Conclusions

Our study found that despite caregivers perceiving that they have good knowledge of asthma and its management, they still identified several desirable educational and interactive features that they wanted to have in a mobile app for asthma self-management. This highlights the potential role that mHealth can play in asthma self-management. Additionally, caregivers were enthusiastic about sharing their data through a mobile app for research. By identifying these preferences and needs, our findings provide a foundation and starting point for co-designing and codeveloping mHealth tools, so that they are relevant to caregivers of children with asthma.

## Abbreviations

CHU: Centre Hospitalier Universitaire
mHealth: mobile health
