# Correction: Caregivers’ Perceptions, Needs, and Data Sharing Concerns in mHealth Research on Pediatric Asthma: Cross-Sectional Survey Study

**DOI:** 10.2196/56046

**Published:** 2024-01-12

**Authors:** Glen Meng, Maliha Jan Ali, Sze Man Tse

**Affiliations:** 1 Faculty of Medicine, Université de Montréal, Montréal, QC, Canada; 2 Division of Respiratory Medicine, Department of Pediatrics, Centre Hospitalier Universitaire Sainte-Justine, Montreal, QC, Canada

In “Caregivers’ Perceptions, Needs, and Data Sharing Concerns in mHealth Research on Pediatric Asthma: Cross-Sectional Survey Study” (JMIR Pediatr Parent 2023;6:e49521) the authors noted one error.

In the original publication, Figure 2 included the correct caption but the image was a reproduction of Figure 1. This has been corrected, and Figure 2 will appear as attached.

**Figure 2.**
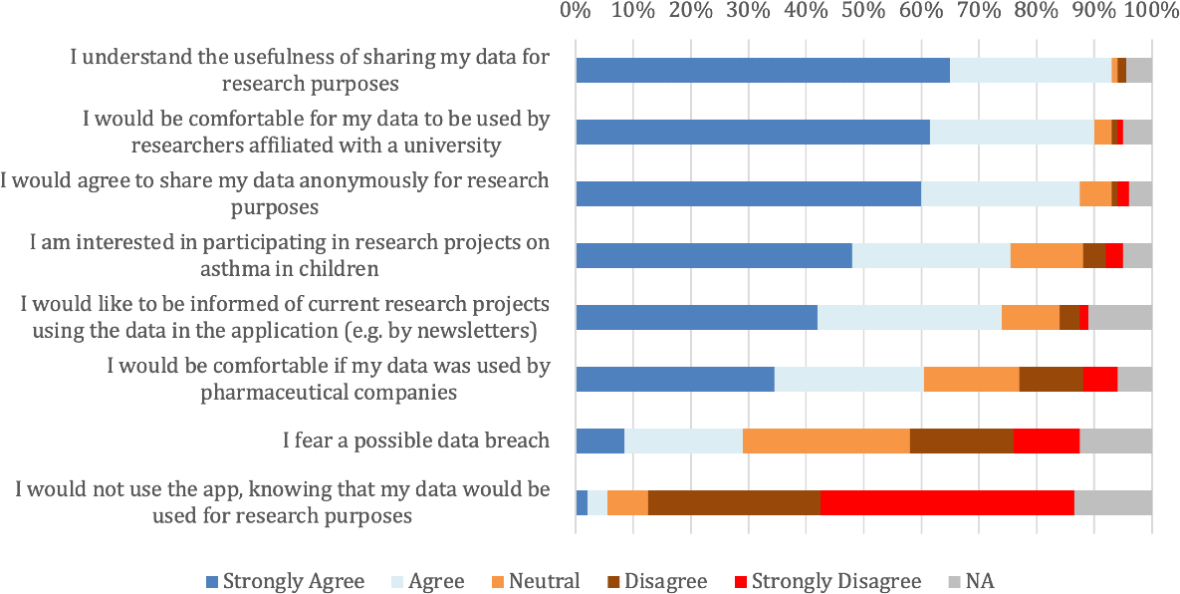
Caregivers’ perceptions on the use of data through mHealth for research. N/A: not applicable.

The correction will appear in the online version of the paper on the JMIR Publications website on January 12, 2024 together with the publication of this correction notice. Because this was made after submission to PubMed, PubMed Central, and other full-text repositories, the corrected article has also been resubmitted to those repositories.

